# Pilot Study to Implement a Resident and Family Decision Guide

**DOI:** 10.1093/geroni/igaa057.2743

**Published:** 2020-12-16

**Authors:** David Wolf, Janet Sopcheck

**Affiliations:** 1 Bellarmine University, Louisville, Kentucky, United States; 2 Florida Atlantic University, Boca Raton, Florida, United States

## Abstract

In this first stage of an 8 state initiative designed to assist nursing homes in reducing unnecessary hospital readmissions, 16 nursing homes were identified and invited by CMS and state agency advisors to participate in the pilot study of the effects of intervention (use of the Guide). Selected facilities received an online orientation to the project and onsite visit from project team leadership prior to launch. Pre and post implementation data were uploaded to a secure section of the project website by the facilities. Three facilities withdrew due to change in top management and a fourth facility provided incomplete data resulting in data for analysis from 12 pilot facilities. Results show the average reduction in readmissions was 31.2% for the project period as compared with the 3-month pre-project period. This presentation will include facility reports of the effect of Guide use on resident and family decision making.

